# The Systemic Changes to Improve Efficiency in Polish Primary Health Care

**DOI:** 10.3389/fphar.2016.00209

**Published:** 2016-07-13

**Authors:** Tomasz Holecki, Piotr Romaniuk, Joanna Woźniak-Holecka

**Affiliations:** ^1^Department of Health Economics and Health Management, School of Public Health in Bytom, Medical University of Silesia in KatowiceBytom, Poland; ^2^Department of Health Policy, School of Public Health in Bytom, Medical University of Silesia in KatowiceBytom, Poland; ^3^Department of Health Promotion, School of Public Health in Bytom, Medical University of Silesia in KatowicePoland

**Keywords:** primary health care, health care reform, efficiency, health management, Poland

## Abstract

Primary health care is an important part of any health care system. In highly developed countries it secures the population's most elementary health needs, with particular emphasis on preventive care and early intervention. Polish PHC model is currently undergoing a thorough transformation, associated with the need to adapt to standards designated based on the WHO's criteria, and with reference to the experience of other European countries. The paper describes the process of changes being carried out, in the context of previous experiences of reform relating to the sphere of organization, processes and efficiency. A review and systematization has been made, with regard to the undertaken activities in the field of deregulation and change of legal provisions, which are aimed at achieving the improvement of the efficiency of treatment and resource allocation. A set of recommendations based on expert's discourse have also been provided, with respect to future directions of Polish PHC transformation.

## Introduction

In developed countries, primary health care (PHC) protects most of the population's health needs, having laid special emphasis on preventive care and early intervention. Similar is its role in Polish health care system, which is however subject to a process of change, including a transition from the model of budgetary financing to a mixed one, inspired by the experiences of other European countries and WHO recommendations^1^.

On the level of primary health care in Poland there are acknowledged physicians of different specialties, not only family medicine specialists, auxiliary staff, which includes nurses, midwives, receptionists, therapists and other medical professionals with different levels of experience. Additionally, health measures in PHC are conducted with the use of patchy technical equipment of facilities, diversified rules of work organization, as well as heterogeneous ownership structure, which includes private providers (currently dominant), along with public and social ones. Inconsistent is also legal environment, since part of the provisions, such as those arising of Labor Code (Ustawa z dnia 26 czerwca, 1974), or the Law on Medical Activity (Ustawa z dnia 15 kwietnia, 2011) applies to all providers, while other laws are applied only to public institutions or those, which have signed a contract with the National Health Fund (NHF) and are obliged to respect the provisions of the Law on Health Services Funded from Public Resources (Ustawa z dnia 27 sierpnia, 2004).

Although many changes have been made in the area of primary care, the satisfying level of efficiency has not been achieved so far, the more that until now, not a single institutionalized tool of measuring the effectiveness of treatment in the primary care has been applied. As a natural consequence—the level of financing of the individual providers has not been connected with its level of efficiency in any way, except for the negligible mechanism of increasing by 1.35% on average of the capitation rates for units ordering, and reporting in details, more laboratory tests (provision enforced in November 2015., and now suspended to equalize rates for all providers). Additionally NHF grant some extra points in the process of contracting services for units having voluntary quality management certificate.

Most importantly—no improve of the effectiveness of treatment, in conjunction with a favorable change in health indicators and cost effectiveness, has been assured, which, to some extent, is a similar phenomenon, as observed in other post-communist countries (Jakovljevic et al., 2016). At the moment, PHC in Poland is experiencing repeating attempts to reform, aimed at improving the rationality of public spending, while taking into account the expectations of patients regarding the availability, quality, comprehensiveness and continuity of care.

Attempts to modify the system of the service provision, according to health care experts should use internationally proven tools to evaluate the effectiveness of treatment, which should then be a basis for remuneration of individual providers. With high probability, however, representatives of providers will harshly criticize this direction of change, as they used to do several times in previous years, when Poland had to face strikes paralyzing the system of primary care. At the same time, the activities of the Ministry of Health and NHF aim to shift further responsibilities to the primary care sector. This is due to financial and organizational constraints, as PHC is the least expensive level of health service provision. The result has so far been some increase in capitation rates, which is the basis for the financing of this level of care, in exchange for the inclusion among PHC tasks new duties related to the oncological treatment (Seifert et al., 2008; Holecki and Romaniuk, 2015), widening the powers of midwives in gynecology and nurses in terms of the ordination of drugs.

This paper refers to the broad context of the functioning of PHC in Poland, consisting of critical analysis of the sources and causes of the current state of affairs. We review the previous reform experiences and their functional consequences. Eventually, based on the latest literature sources and a review of official acts, we outline the likely scenario of further changes.

## Systemic causes of low efficiency of PHC in Poland

Among the most primordial reasons for the insufficient organizational capacity of PHC in Poland, there are problems of mentality, ingrained in the awareness of all stakeholders responsible for the shape and functioning of the health system. That problem arises from many years of experience of communism and habits that grown of Semashko model implemented in Poland's health system after the Second World War. This paradigm remained a basis for the organization of the system actually until the very end of the Twentieth century. Among the elements that characterized this model was a domination of hospital treatment, based on an extensive infrastructure and high prestige of doctors of clinical specialties. This was additionally strengthened with free access to specialists, not requiring a patient to have a referral from general practitioner, who, in such circumstances, had been perceived as a doctor of inferior significance, useful only in case of minor health issues.

Funding was carried out on the basis of the estimated global budgets, in isolation from the criteria of efficiency. Additionally, until 1999 patients were secured with access to PHC based on their place of residence, having no right to choose provider, which eliminated any possibility of developing mechanisms of competition (Tomasik et al., 2013). Moreover, what it is typical for all the economies operating under a deficit, the model has developed a system of socially tolerated corruption. This became one of the causes of failure of reforms in the last decade of the Twentieth century, where reform leaders had to deal not only with the objective constraints, like the lack of funds for maintenance and development of the system, or the general turbulence of the economy in transition, but also the reluctance to change. Until now in common use there is a term “health service,” assuming a disinterested and non-for-profit help to another person, which is opposed to the concept of “health care market” as a commercial solution, and thus exclusive and socially unjust.

Conceptual schemes ingrained in the minds of health system participants, connected with the lack of political determination, complexity and consistency in the implementation of the assumed solutions, resulted in preservation of the specific state of inertia. It is also worth noting that the educational gap characteristic for the post-communist society, which manifested itself particularly strongly in a deep ignorance of managerial mechanisms and basic market rules, contributed to the organizational failure of the whole health sector, which for years has been fitted into a “developmental drift.”

Another cause of indolence of Polish PHC must be clearly emphasized against the rules of political correctness. It is the insufficient level of competence of primary care physicians, in conjunction with staff shortages. This is a clear derivative of the system imperfections, particularly the difficulties in getting access to specialty trainings. This, in turn, results of the caste structure deeply rooted in the medical community. A consequence of this specific mechanism of professional exclusion was a situation, where whole groups of young medical professionals were pushed out to the margins of the health care system.

The first real attempt to reform the system have been taken in the 90's of the Twentieth century. The assumption was to transform primary care into a British-like model (Krztoń-Królewiecka et al., 2013). A new specialty in family medicine had been implemented, assumed to become commonly popular (Przekształcenia podstawowej opieki zdrowotnej. Strategia realizacji leków, 1994). Young doctors were proposed to achieve specialization under a three-year residency. First such a training program was launched in 1994. For the experienced PHC doctors, a so-called “short educational path” had been offered, lasting for only 6 months. These activities were supported by World Bank's and pre-accession EU funds, and their effect was education of nearly 5000 family medicine specialists, up to the end of 2000 (Kosiek, 1997). This policy temporary filled the employment gap, but no guarantee of the persistence of the trend was ensured, as the acquired specialization was perceived as of low prestige, and did not translate into wage growth. Consequently, the issue of staff shortage is still a problem, which in turn results in an excessive work burden on doctors. This is confirmed by the official upper limits of patients per one general practitioner in Poland, which are the highest in CEE region (Oleszczyk et al., 2012). This, in turn, has a negative impact on doctors' psycho-physical work conditions, as well as reduces the quality of services and causes a negative perception of the PHC system by patients (Coulter and Jenkinson, 2005).

In financial terms, almost entire accounting system used in PHC is based on capitation rate, which is a monthly fee for the care of the group of patients declared to the doctor, nurse and midwife. Such a solution significantly simplifies the remuneration system, but at the same time deprives the positive financial incentives and strengthens the mechanism of pushing patients to specialist care.

## Current reforms in PHC in Poland

It is unacceptable to keep tolerating the lack of uniform standards, effective motivational tools and clear criteria for evaluation of medical staff in Polish PHC. In case of further avoidance of adequate solutions, in conjunction with the demotivating factors and limitations of the system, the system will experience further persistence of the low service quality (Tomasik et al., 2013), late, inadequate and incomplete response of general practitioners to changing guidelines for the treatment of certain diseases, and significantly lower effectiveness of provided treatment.

An objective for decision makers initiating the change should be to create effective systemic mechanisms, which would allow not only a diverse valuation of benefits in connection with their actual quality, but also to facilitate choices made by individual patient. The development and application of standardized assessment tools should be the first step toward improving the quality of services. Currently, despite declarations, no major standardization tools, or measurements of the patients' satisfaction and effectiveness of treatment have been applied. The only exceptions are voluntary instruments linked to the implementation of the quality management systems.

The applied rules of contracting services are definitely outdated and inadequate, not only to the needs but also to the existing possibilities. Capitation method is an effective mechanism only for cost containment (Kowalska et al., 2015), having no impact on the efficiency of treatment. At the same time it intensifies the efforts of providers and doctors to maximize their own income, which is achieved by increasing the number of patients declared with a doctor, along with restrictions on access to diagnostic services or shifting costs to the sector of specialist care.

In financial terms, we observe insufficient resources combined with their incorrect allocations. In this area, there is also the issue of the lack of mechanisms connecting the amount of funds for individual provider with his operational effectiveness. Adopted solutions disable the PHC physicians from taking the assumed role of coordinator of the process of treating their patients, being rather an incentive to reduce their own responsibility than to increase it. Existing solutions do not address factors encouraging general practitioners to systematically improve their professional qualifications, which in practice leads to a depreciation of their competence.

Currently PHC in Poland is in a situation of waiting for a thorough change, the scope of which is to cover three dimensions of its operation: the structure, processes and results. In January 2016, on behalf of the Ministry of Health, a preparations of the project of complex changes has started. A panel of experts, including representatives of all organizations of family doctors, is expected to prepare the assumption of the new law until June 2016, along with the PHC development plan for further years. Members of the panel proposed a set of possible solutions, like remuneration for the effects. In such a case the capitation rate would represent only part of the PHC providers incomes (as assumed—80% at a maximum). The remaining part would be dependent on keeping patients in good health, or applying a widely defined preventive measures, which currently is strongly neglected. Among the likely indicators to be used, there are: fasting blood glucose or glycated hemoglobin (HbA1c) in patients with diabetes, targeted blood pressure or cholesterol in patients with cardiovascular diseases, body weight reduction in patients with obesity and overweight. In case of smokers, number of patients, who dropped permanently smoking might be awarded, as verified during the period of half a year, then the year and two. In general, indicators should relate to common diseases and measure the effects dependent on medical action at this level of care. Two other newly established panels should simultaneously work on changes related to deregulation in health system, and general remodeling of the health system^2^.

The existing model of separate contracting of PHC doctor, nurse and midwife is to be replaced with the cumulative contract for the whole team under the supervision of a physician. It is also envisaged to supplement those teams with a psychologist. This contracting proposal met a very strong protest of nurses and midwives associated in professional self-government organizations. Supreme Council of Nurses and Midwives is in a position that the adopted proposals infringe the principle of economic freedom, as expressed in the Constitution (Konstytucja Rzeczypospolitej Polskiej z dnia 2 kwietnia, 1997). In particular, the proposal to create “medical and nursing teams” with the role of the physician as a primary care coordinator, and the lists of patients declared commonly to a doctor, nurse and midwife is being criticized. According to nurses and midwives, new solutions represent a threat to their professional independence (Ustawa z dnia 15 lipca, 2011). This opinion is so justified, that the current project is contrary to recent amendments, which gave nurses and midwives, in case of having an appropriate higher education of master's degree or specialization, a permission to independently prescribe medicines containing certain active substances and foods for particular nutritional uses. According to the law, this applies to some weaker drugs, the list of which has been defined in the Regulation of the Minister of Health of 20th October 2015 (Rozporządzenie Ministra Zdrowia z dnia 20 października, 2015). The list consists of 31 substances in 16 groups, e.g., antiemetics, different types of anti-infectives, anesthetics or painkillers. Furthermore, the proposal of common contracting and joint declaration of provider choice is inconsistent with the Law on Health Services Funded from Public Resources (Stanowisko nr 4, Naczelnej Rady Pielęgniarek i Położnych z dnia 8 marca, 2016).

A place of permanent dispute on competences and financial issues is also PHC cooperation with hospitals. Representatives of the inpatient sector are protesting against embarking them with a large part of the duties of PHC, which appears e.g. during the weekend and night duties, as PHC sector provides services only from Monday to Friday from 8.00 to 18.00. In such a situation, they believe increased PHC funding is unfair and needs to be corrected at the level of coordination between all elements of the health system.

Regardless of the announcement of thorough regulations of the issues of family medicine, the first minor changes are already being implemented. Regulations enforced by the NHF in 2015 (Zarządzenie nr 77/2015/DSOZ Prezesa NFZ z dnia 19 listopada, 2015), assuming better remuneration of doctors who prescribe more additional diagnostic tests, has been abandoned. Currently, a higher capitation rate will be paid to all doctors, and the reporting on selected specialized examination has been changed from quarterly to semi-annual, to be soon completely abolished. Nurses and midwives since January 2015 received wage growth, by addressing the PHC sector with a stream of “labeled” money just for this purpose from the regional NHF branches. Further salary increase have been announced for the next three consecutive years. While undoubtedly salary increase for the least paid PHC workers is a positive action, it is clear that this kind of central control deprived the health facilities managers of decisional abilities, although formally they are autonomous in decision making. Additionally the wage regulation omitted other PHC labor groups, like the receptionists or office nurses.

Improvements has been also announced in relation to the oncological package. The Ministry of Health proposes to replace the paper version of the card of oncological diagnosis and treatment (DiLO) with the sole electronic version. Additionally sanctions for over-sized level of incorrect cancer diagnoses are to be abandoned. Currently, if general practitioner refers a patient to the so-called “oncological fast track,” and cancer is confirmed in less than 1 in 15 people, the doctor is excluded from the possibility of writing more such referrals. This type of punishment, along with the obligation to pass training courses for physicians, is not only a kind of stigma in the professional environment, but also gives rise to a number of risks associated with defensive behaviors when planning of oncological treatment.

The proposed deep changes in Polish PHC, which are being encouraged by the Minister of Health, on the one hand are strongly expected by experts and service providers, especially in the area of deregulation and simplification of existing rules, in particular those relating to the extensive reporting. On the other hand, although they are a chance to improve the effectiveness of treatment and management, they also conceal the seeds of conflict within the environment.

## Conclusions

Reorganization of primary health care in Poland coordinated by the Ministry of Health involves efforts of many groups related to health care—the representatives of ministries and public payer, representatives of professional associations, providers associations, and organizations representing the interests of patients. An interdisciplinary panel of experts has been appointed at the beginning of 2016 to prepare a strategy for systemic solutions in PHC, which are the basis for further legislative changes (Zarządzenie Ministra Zdrowia z dnia 4 stycznia, 2016). As a result of the panel's work, on 5th April 2016 a draft of the amendment to the Law on Health Services Funded from Public Resources, the Law on Pharmaceutics (Ustawa z dnia 6 września, 2001), as well as the Law on the prevention and elimination of infections and communicable diseases (Ustawa z dnia 5 grudnia, 2008), has been sent for public consultation.

It should be clearly emphasized, that these actions must be conducted in a strategic perspective, and not just *ad hoc*, which requires reformers not only to have knowledge and commitment, but, above all, a comprehensive look at the entire health care system. This is important also because the initiated changes will penetrate extremely sensitive area of social life, centered on the human and civil rights to health.

According to the Spokesman for the Rights of Patients, PHC is especially lacking a complex, well prepared and consistently applied prevention, which is confirmed by the report of the Supreme Chamber of Control (Supreme Audit Office, 2015) showing that more than a half of the inspected providers not carried out their tasks in accordance with the range originally assigned to the PHC. Among the postulates submitted by family doctors, particularly important is long-awaited increase of their role in the system, followed by clear formal regulations and better financing. Public health market experts in turn to pay particular attention to the potential difficulties in developing and implementing a coherent model of PHC organization and financing, yet so universal to be suitable for all providers, in particular to improve coordination of care and cooperation between physicians, nurses and midwifes (Primary Health Care, 2016).

In addition, it should be remembered that the rebuilt PHC model is not only a component of the national health system, but also fits the coordinated health care of the European Community^3^, particularly with regard to the provisions of the Directive on cross-border health care^4^. Thus, the actions and tools taken from other health systems to improve efficiency should lead to sustained optimization of the provision of PHC services, which is currently devoid of any serious tools of organizational and financial motivation.

## Author contributions

TH conceived the study and prepared draft of the paper and made the study. PR contributed to paper preparation and study. JW contributed to paper preparation and study.

### Conflict of interest statement

The authors declare that the research was conducted in the absence of any commercial or financial relationships that could be construed as a potential conflict of interest.

## Footnotes

^1^WHO Conferences: Ałma-Ata 1978, Bordeaux 1983, Copenhagen 1991, Copenhagen, 1999.

^2^www.sluzbazdrowia.com.pl (Accessed: 8 May 2016); www.medexpress.pl (Accessed: 5 May 2016).

^3^Regulation (ec) No. 883/2004 of the European Parliament and of the Council of 29 April 2004 On the coordination of social security systems.

^4^Directive 2011/24/EU of the European Parliament and of the Council of 9 March 2011 On the application of patients' rights in cross-border healthcare.
